# Associations of Occupational, Socio-Demographic and Lifestyle Factors with Lung Functions in Malaysian Traffic Policemen

**DOI:** 10.5334/aogh.2895

**Published:** 2020-07-28

**Authors:** Putri Anis Syahira Mohamad Jamil, Karmegam Karuppiah, Irniza Rasdi, Vivien How, Shamsul Bahri Mohd Tamrin, Kulanthayan K. C. Mani, Sivasankar Sambasivam, Nur Athirah Diyana Mohammad Yusof, Nurul Maizura Hashim

**Affiliations:** 1 Department of Environmental and Occupational Health, Faculty of Medicine and Health Sciences, Universiti Putra Malaysia, Selangor, MY

## Abstract

**Background:**

Apart from being exposed to various hazards, there are several other factors that contribute to the deterioration of traffic police health.

**Objectives:**

A cross-sectional study was carried out to explore the association of occupational, socio-demographic, and lifestyle factors with lung functions in traffic policemen in Kuala Lumpur (KL) and Johor Bahru (JB).

**Methods:**

A spirometer was used to measure lung function of subjects, whereas a self-administered questionnaire was used to obtain their information on background data, lifestyle, and occupational factors. The statistical test used was Spearman rho’s test and chi-square test; then, the factors were further tested using Logistic regressions.

**Findings:**

134 male subjects were selected as respondents in this study with 83% response rate. Among all the factors tested, age (FVC: χ = 8.42(3), p = 0.04), (FEV: χ = 8.26(3), p = 0.04), rank (FVC: χ = 8.52(3), p = 0.04), (FEV: χ = 8.05(3), p = 0.04), duration of services (FVC: χ = 11.0(1), p = 0.04), (FEV: χ = 6.53(1), p = 0.01), and average working hours (with the Measured FVC (litre), r = –3.97, p < 0.001; Measured FEV1 (litre), r = –3.70, p < 0.001; Predicted FVC, r = –0.49, p < 0.001; Predicted FEV1, r = –0.47, p < 0.001; and %Ratio FEV1/FV, r = –0.47, p < 0.001) were significantly related to lung function among traffic police.

**Conclusions:**

Occupational factors play a crucial role, and hence, the authorities should take action in generating flexible working hours and the duration of services accordingly. The data from this study can help by serving as a reference to the top management of traffic police officers to develop occupational safety and health guideline for police officers to comply with the Occupational Safety and Health Act (OSHA, Act 514 1994).

## Introduction

Since the Malaysian traffic police in the Point Duty Department is working in an outdoor environment for long and irregular hours, they are directly exposed to air pollutant scattered in the air. The traffic police were exposed to polluted air more frequently as their duties adjure them to control road traffic at the highly congested junctions [1]. Under the Police Act, 1967 Section 21 task of regulating, monitoring, and maintaining the flow of traffic on public roads falls to the responsibilities of the traffic policeman [2]. With such obligations; they have no choice other than to perform the given task. Their task was considered heavy duty as they had to deal with congested traffic condition and attitude of selfish drivers. Kuala Lumpur (KL) and Johor Bahru (JB) primarily is a crucial workplace for traffic police since both areas serve as the busiest city centres in Malaysia with many industrial and business operations [3]. In 2016, the number of vehicles reported was already 6.27 million in KL, 3.58 million in JB and the number is rising each year [4].

Those polluted air can cause respiratory irritation or breathing difficulties, even for healthy people. The actual risk depends on current health status, the pollutant type and concentration, and the length of exposure to the polluted air [5]. According to Dutta T and Pal G [6], statements, long term exposures to gases and fumes present in the environment near heavy traffic decreases the lung functions. Coupled with high concentrations of air particles, this can lead to respiratory diseases or have already caused respiratory symptoms among those exposed to them [7]. In occupational respiratory disease, measurements of lung functions are obtained by using spirometry as it is one of the most important diagnostic tools. It is also used for screening workers with exposures to agents associated with lung diseases [8]. Benefits of using this test are that it provides a clearer understanding of lung function in subjects of different races, age, sex, occupation, and profession, as stated by Mariammal et al. [9].

Aside from exposure to traffic-related air pollution, lung function is influenced by other factors such as gender, age, height, weight, ethnicity and other activities [10]. Several studies among traffic police personnel have reported declines in their lung function associated with exposure to traffic-related air pollution, especially in India, China, and Thailand. In contrast, the research is very limited in Malaysia. With regards to the other possible associated factors towards lung function among traffic police personnel, there is not much reporting on this. Specifically, there was no study has been conducted in traffic policemen in KL and JB.

In response to this problem, this study proposes to investigate the association of occupational factors, socio-demographic, and lifestyle with lung functions in traffic policeman who works in dense traffic areas in KL and JB.

## Materials and Methods

### Study Location

This cross-sectional study was conducted from the end of January 2015 until end of June 2015. As advised by Bukit Aman Headquarters, the study location was chosen, using purposive sampling. To have a more significant collection of data, the researchers included a few police stations. This study was conducted in Traffic Branch Police Station in KL (Balai Trafik Jalan Tun H.S. Lee) and Johor (Balai Trafik Johor Bahru). These Traffic Branches have the highest number of traffic policemen working in Point Duty Unit, which is the target respondents for this study. Traffic police in the Point Duty Unit mainly work on regulating the traffic flow in congested junctions all around the city centre and outskirts [11]. According to Bukit Aman Headquarters, at times of data collection, there were 146 traffic police in Point Duty Unit in KL Branch, whereas, in Johor Bahru Branch, there were 77 traffic police working in Point Duty Unit. Police stations in other states do not have Point Duty Unit as the demands for traffic control is not as high as in KL and Johor Bahru. However, when needed, traffic police from other units from the same police stations will be deployed to carry out traffic control on the road. Since KL and Johor Bahru had similar situations regarding their traffic volume, the research is done in these states. Also, the only Traffic Branch is located in these states as the needs of traffic police is high in these areas. By looking at the air pollution index in 2015 (at the time of data collection), both locations were in unhealthy status, which was 170 for KL and 198 for JB. The status is 83 overwhelming for both states, whereby everyone may begin to experience health effects with 84 members of sensitive groups may experience more severe health effects. Moreover, the number of traffic police riders in KL and Johor was the largest in Malaysia [11].

### Study population and Inclusion Criteria

The study population was the traffic policeman of the Point Duty Unit in Malaysia. The sampling unit has met the inclusion criterion, which is a healthy traffic policeman in the age group of 20–56 years who are working in traffic junctions for more than one year. The range of age group was decided by merging a range of age people working in Malaysia and the range of group according to the reference value for lung function test [12] so that their predicted value for lung function parameters was able to determine. Plus, the traffic police worked for more than one year were identified and involved in the study so that they were adapted to their nature of work for consistency of data. The exclusion criteria were traffic policemen with respiratory diseases such as asthma, bronchitis, and wheezing, as well as those who are not willing to participate in this study. The exclusion criteria were to ensure that the possible participants of this study are having the respiratory symptoms due to the study factors, not by hereditary factors or already developed diseases.

### Sample Size

The sample size calculation was based on findings of [13] on exposure to PM_2.5_ and lung function among traffic police and general police in KL, which reported a significant reduction in all lung function parameters (p < 0.05). Thus, the mean and standard deviation were used in a formula as a reference. The formula used to determine the sample size is by using Lemeshow [14] formula shown below:

{\rm{n}} = \frac{{2{\sigma ^2}{{\left[ {{Z_{1 - \frac{a}{2}}} + {Z_{1 - \beta }}} \right]}^2}}}{{{{\left({{\mu _1} - {\mu _2}} \right)}^2}}}

{\rm{n}} = \frac{{2{{\left({0.88} \right)}^2}{{\left[ {1.96 + 0.842} \right]}^2}}}{{{{\left({4.14 - 4.51} \right)}^2}}}

n = 88.70 ≈ 89 in each group

n = 89 respondents per group × 2

n = 178 respondents total

Where:

**σ** = standard deviation in FVC is estimated at 0.88 (assumed to be the same for both groups).

**µ_1_** = mean of FVC among a low concentration of PM_2.5_ at 4.14 litre

**µ_2_** = mean of FVC among a high concentration of PM_2.5_ at 4.51 litre

Hence, the total number of respondents needed in this study is 178. However, according to Aday and Cornelius [15], additional adjustments were made to ensure the required target number of sample size is obtained. Thus, the adjustment for the size of the population, expected response rate, and the expected proportion eligible for sample size had been made based on the beginning sample size of 178 respondents.

**Table:** 

i.	Adjust for the size of the population:	
	n = n/(1+(n–1)/N)	
	n = 178/(1+(178–1)/356	
	n = 119	
	where n = 178; N = 119	
ii.	Adjust for the expected response rate:	
	The response rate is 0.80	
	therefore n = 119/0.80	
		n = 149
iii.	Adjust for the expected proportion eligible:	
	% Eligible = 0.95	
	therefore n = 149/0.95	
		n = 157

After the additional adjustment has been made, the total sample size for this study is 157 respondents. However, the total response rate was 85.35% (N = 134) and was acceptable, according to Richardson [16], which stated that an acceptable response rate for social research was 50%. The lack of participants was due to drop out as they did not fulfill the inclusion criteria after being recruited and the unfortunate things that happen, such as involved with accidents at work.

### Sampling Method and Data Collection

**Figure 1:**
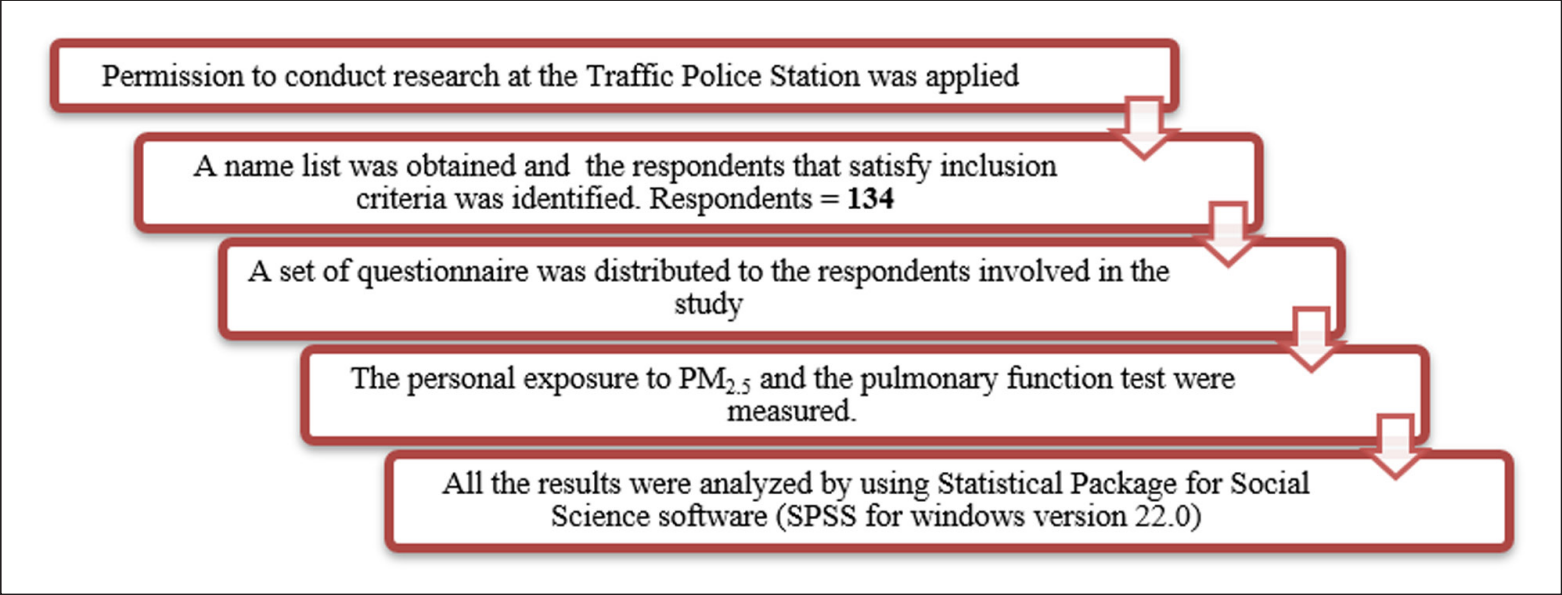
The flow chart of the research procedure.

The sampling method was simple random sampling, where the respondents were selected based on the inclusion criterion. Firstly, permission to research the Traffic Police Station was applied, and the name list was obtained. Later on, the questionnaires were distributed to respondents that fulfill the inclusion criteria. At the start of their day, the air sampling pump was given to them and wore throughout the day until the end of their shift. Lung function test was done at 10 a.m. in a specified room given by the Officer in Charge of the police station, as they will come back to the police station for briefing. The research procedure was illustrated in Figure 1, as shown below.

The inter-observer bias was controlled by ensuring all the procedures were standardised. For example, the lung function test was made in a similar office room throughout the study without any disturbance and audience. The next respondent was placed in the waiting room next to the test room. The standard procedure of lung function test, ATS/ERS Standardisation of Spirometry was followed, and to control experimenter effects, a second researcher was placed inside the room to monitor the test.

### Study Instrumentations

To obtain accurate data on body weight and height, the researchers used SECA products (SECA Body Meter and SECA Body Weighting). SpirolabIII by Medical International Research were used to measure the lung function status of respondents. Standard procedures of lung function test, which is adapted based on ATS/ERS Standardisation of Spirometry [17], was used in this study. The predicted value was obtained by using reference equations 1 and 2 for Malaysian by Singh et al. [12], which studies on spirometric between 13 and 69 years of age. The reference equation is: For male: Age (20–69 years old)

1 {\rm{FVC}}\; \left({{\rm{litre}}} \right) = 0.0407 \left({{\rm{Height}}} \right) + 0.0296 \left({\rm{Age}} \right) - 2.343

2 {\rm{FEV1}}\;\left({{\rm{litre}}} \right) = 0.0353 \left({{\rm{Height}}} \right) + 0.0315 \left({{\rm{Age}}} \right)

Socio-demographic and lifestyle of respondents were obtained by using a self-administered questionnaire. This questionnaire was used to know the background information and general health status. The form was used in Bahasa Malaysia (National Language for Malaysia) so that the respondents can easily understand the question given. The questionnaire had undergone constructive testing and reliability testing. After conducting the pre-test, the questionnaire had acceptable reliability (Cronbach’s α = 0.92). There were four main parts of this questionnaire. Background information such as age, race, height, and weight were asked in the first section (section A) in the questionnaire. Section B, C, and D determined their history of occupation, health information, and lifestyle, respectively.

### Data analysis

Data collected were analysed using univariate, bivariate, and multivariate analysis. Lung function test and other associated factors were analysed by using Statistical Package for Social Science (SPSS version 22.0). This study was conducted using 80% of power, 95% confidence interval in which a result of p < 0.05 was considered significant. The descriptive test was used to produce mean and standard deviation value for the respondent’s background information and other variables. Since the data were not normally distributed, the chi-square test was used to determine the association between risk factors and lung function. After testing for bivariate analysis, the factors that show significance was further tested using Logistic regressions. From this, the formula for predicting the prevalence of lung function was constructed.

## Results

A total of 134 male subjects were selected as respondents with an 83% response rate due to the hectic work and mobile as traffic police and also their work nature that makes it difficult to achieve 100% of respondents. During their short ample time, they were required to fill in the questionnaires that were given to them. Tables 1, 2, and 3 show the characteristics of the study population in descriptive. Referring to Table 1 below, the mean age for both study location are 35 years old (*SD* 10.1). Besides, the majority of the respondents were Malay (65.7% and 87.5%), followed by others (30% and 12.5%). Meanwhile, Indian (1.5%) and Chinese (0.7%) respondents were obtained only from KL police station. The majority of the respondents had completed Secondary education (80% and 93.8%), followed by Tertiary education (20% and 4.7%), and Primary education (1.6%) respondents were obtained only from JB police station. A total of 76.9% of respondents were married, and 23.1% are single. In this study, the mean height of respondents in KL and JB was 170.3 cm (*SD* 4.8) and 170.7 cm (*SD* 6.1), respectively; meanwhile, the total mean height was 170.5 cm (*SD* 5.4). The mean weight of respondents in KL was 72 kg (*SD* 10.3), and JB was 76.1 kg (*SD* 13.9); meanwhile, the total mean weight was 74 kg (*SD* 12.5).

**Table 1:** Characteristics of the study population, N = 134.

Variables	KL		JB		Total	

	F (%)	Mean ± SD	F (%)	Mean ± SD	F (%)	Mean ± SD

**Age**						
20–30 years old	18(25.7)		14(21.9)		32(23.9)	35 ± 10.1
31–40 years old	25(35.7)		13(20.3)		38(28.3)	
41–50 years old	13(18.6)		15(23.4)		28(20.9)	
≥51 years old	14(20)		22(34.4)		36(26.9)	
**Race**						
Malay	46(65.7)		56(87.5)		102(76.1)	
Chinese	2(2.9)		0		2(1.5)	
Indian	1(1.4)		0		1(0.8)	
Others	21(30)		8(12.5)		29(21.6)	
**Height (cm)**	70(100)	170.3 ± 4.8	64(100)	170.7 ± 6.1	134(100)	170.5 ± 5.4
**Weight (kg)**	70(100)	72.0 ± 10.3	64(100)	76.1 ± 13.9	134(100)	74.0 ± 12.5
**Educational level**						
Primary	0		1(1.6)		1(0.7)	
Secondary	56(80)		60(93.7)		116(86.6)	
Tertiary	14(20)		3(4.7)		17(12.7)	
**Marital Status**						
Single	26(37.1)		5(7.8)		31(23.1)	
Married	44(62.9)		59(92.2)		103(76.9)	
**Total**	**70**		**64**		**134**	

As shown in Table 2, 81.3% were smokers, and despite their hectic life in working as traffic police, 82.8% of them were involved in any physical activities which they did during their ample time.

**Table 2:** Lifestyle characteristics on the subject, N = 134.

Variables	KL		JB		Total	

	F	(%)	F	(%)	F	(%)

**Smoking habits^a^**						
Yes	57	81.4	52	81.3	109	81.3
No	13	18.6	12	18.7	25	18.7
**Physical activity^b^**						
Yes	59	84.3	52	81.3	111	82.8
No	11	15.7	12	18.7	23	17.2
**Total**	**70**		**64**		**134**	

^a^ smoking habit: a physical addiction to tobacco products. Source: Encyclopedia of Children’s Health. In this study, respondents who have experience smoking pipes, cigarettes, or cigars throughout their life would answer yes to the question. ^b^ physical activity: any body movement that works our muscles and requires more energy than resting (e.g. aerobic, muscle-strengthening). Source: National Heart, Lung, and Blood Institute, United States. In this study, respondents with weekly routine physical activity, whether its work out or jogging, as long as it is outside of work.

Table 3 shows According to rank, the majority of them were Corporal (24.3% and 59.4%), Constable (52.9% and 7.8%), Lance Corporal (15.7% and 21.9%), and the least were Sergeant (7.1% and 10.9%). By looking at the table below, the mean duration of services in the traffic department was 6.5 years (*SD* 5.0), whereby in total, 53.7% of the respondents have worked for less than six years, and 47.3% have worked for more than six years in this department. The traffic police in this study have an average working hour of 9.5 hours (*SD* 3.0) per day. From the questionnaire, none of them wore any personal protective equipment such as a mask.

**Table 3:** Occupational factors on the subject, N = 134.

Variables	KL		JB		Total	

	F (%)	Mean ± SD	F (%)	Mean ± SD	F (%)	Mean ± SD

**Rank**						
Constable	37(52.9)		5(7.8)		42(31.3)	
Lance Corporal	11(15.7)		14(21.9)		25(18.7)	
Corporal	17(24.3)		38(59.4)		55(41)	
Sergeant	5(7.1)		7(10.9)		12(9)	
**Duration of services**						
<6 years	52(74.3)		20(31.2)		72(53.7)	6.5 ± 5.0
≥6 years	18(25.7)		44(68.8)		62(46.3)	
**Average working hours**	70(100)	8.8 ± 2.4	64(100)	10.2 ± 3.3	134(100)	9.5 ± 3.0
**Total**	**70**		**64**		**134**	

The respondents were asked to perform a spirometry test to measure their lung function status. Results, as shown in Table 4 were 91.0% of the respondents recorded low FVC% predicted, and 94% of them recorded low FEV1% predicted, which indicates the abnormality of lung function is high among the respondents of this study. Since there were two different study locations involved in this study, lung function parameters were tested for confounding factors. As shown in Table 4, there was no significant difference detected. These results show that the study locations were not a confounder for lung function in this study.

**Table 4:** Lung function status of the traffic policeman.

Variables	Status	Study Locations Frequencies (%)		Total N = 134 Frequencies (%)	χ^2^ value	p-value

		KL n = 70	JB n = 64			

FVC% predicted	Abnormal	61(87.1)	61(95.3)	122(91.0)	2.74	0.10
	Normal	9(12.9)	3(4.7)	12(9.0)		
FEV1% predicted	Abnormal	65(92.9)	61(95.3)	126(94.0)	0.36	0.55
	Normal	5(7.1)	3(4.7)	8(6.0)		

*chi-square test.

To identify the associations between socio-demographic characteristics and lung function, the researchers conducted a series of preliminary analyses. A significant positive association was found between age (p = 0.04), rank (p = 0.04), and duration of services (p = 0.01) with lung function. Participants with older age and higher rank who serves longer in the Royal Malaysian Police tend to show higher levels of abnormalities in lung function. Apart from this, none of the remaining analyses was significant, as shown in Table 5.

**Table 5:** Association between socio-demographic characteristics, lifestyle and occupational factors with pulmonary function among traffic policemen (N = 134).

Variables	FVC (litre)				FEV1 (litre)			

	Normal	Abnormal	χ^2^	p-value	Normal	Abnormal	χ^2^	p-value

	n(%)	n(%)			n(%)	n(%)		

**Age**								
20–30 years old	12(85.7)	56(46.7)			8(100)	60(47.6)		
31–40 years old	2(14.3)	28(23.3)	8.42	*	0	30(23.8)	8.26	*
41–50 years old	0(0)	21(17.5)	(3)		0	21(16.7)	(3)	
≥51 years old	0(0)	15(12.5)			0	15(11.9)		
**Race**								
Malay	7(50)	95(79.2)			5(62.5)	97(77)		
Chinese	0	1(0.8)	7.56	NS	0	1(0.8)	1.39	NS
Indian	0	2(1.7)	(3)		0	2(1.6)	(3)	
Others	7(50)	22(18.3)			3(37.5)	26(20.6)		
**Educational level**								
Primary	0	1(0.8)			0	1(0.8)		
Secondary	13(92.9)	103(85.8)	0.57	NS	8(100)	108(85.7)	1.32	NS
Tertiary	1(7.1)	16(13.3)	(1)		0	17(13.5)	(1)	
**Marital Status**								
Single	6(42.9)	25(20.8)	3.42	NS	2(25)	29(23)	0.02	NS
Married	8(57.1)	95(79.2)	(1)		6(75)	97(77)	(1)	
**Smoking habits^a^**								
Yes	7(50)	78(65)	1.22	NS	4(50)	81(64.3)	0.66	NS
No	7(50)	42(35)	(1)		4(50)	45(35.7)	(1)	
**Physical activity^b^**								
Yes	102(83.6)	9(75)	0.57	NS	105(83.3)	6(75)	0.37	NS
No	20(16.4)	3(25)	(1)		21(16.7)	2(25)	(1)	
**Rank**								
Constable	9(64.3)	33(27.5)			6(75)	36(28.6)		
Lance Corporal	1(7.1)	24(20)	8.52	*	0	25(19.8)	8.05	*
Corporal	4(28.6)	51(42.5)	(4)		2(25)	53(42)	(4)	
Sergeant	0	12(10)			0	12(9.5)		
**Duration of services**								
<6 years	10(71.4)	51(42.5)	11.0	***	7(87.5)	54(42.9)	6.53	**
≥6 years	4(28.6)	69(57.5)	(1)		1(12.5)	72(57.1)	(1)	

^a^ smoking habit: a physical addiction to tobacco products. Source: Encyclopedia of Children’s Health. In this study, respondents who have experience smoking pipes, cigarettes, or cigars throughout their life would answer yes to the question. ^b^ physical activity: any body movement that works our muscles and requires more energy than resting (e.g. aerobic, muscle-strengthening). Source: National Heart, Lung, and Blood Institute, United States. In this study, respondents with weekly routine physical activity, whether its work out or jogging, as long as it is outside of work. *P < 0.05, **P < 0.01, *** P < 0.001; NS, not significant.

To test the hypothesis that average working hours would be associated with the lung function of traffic police, Spearman rho’s test was conducted. Average working hours was significantly associated with the Measured FVC (litre), Measured FEV1 (litre), Predicted FVC, Predicted FEV1 and %Ratio FEV1/FVC, r = –3.97, p < 0.001; r = –3.70, p < 0.001; r = –0.49, p < 0.001; r = –0.47, p < 0.001; r = –0.47, p < 0.001, respectively. The result shows that the longer the working hours, the lower the value of Measured FVC (litre), Measured FEV1 (litre), Predicted FVC, Predicted FEV1 and %Ratio FEV1/FVC which indirectly indicates an abnormality in their lung function as tabulated in Table 6.

**Table 6:** Relationship between occupational factor with lung function (N = 134).

Variables	Measured FVC (litre)		Measured FEV1 (litre)		Predicted FVC		Predicted FEV1		%Ratio FEV1/FVC	

	r value	p value	r value	p value	r value	p value	r value	p value	r value	p value

**Average working hours**	–3.97	***	–3.70	***	–0.494	***	–0.473	***	–0.474	***

#Spearman correlation. ***Significant at p < 0.001.

For multivariate analysis, after controlling for confounders, there was a statistically significant regression between duration of services (years) and lung function, as shown in Table 7 (p-value < 0.05).

**Table 7:** Factors associated with lung function (FVC) among traffic policemen from logistic regression.

Variables	FVC			

	Coefficient (B)	Adjusted OR (CI)	Wald Statistics (df)	p-value

**Duration of services**				
<5 years	–1.866	0.155	4.836 (1)	*
≥5 years		(0.03–0.76)		
Constant (0.715)				

*Significant at p < 0.001.

## Discussion

Findings from this study show that the abnormality of lung function status is high among traffic policemen tested. Their lung function status should be a vital issue to be addressed soon. This finding is parallel with the previous study [13] in a similar setting, which reported significant depletion in all parameters of lung function among traffic policemen.

In line with the hypotheses, the study demonstrates an association between age and rank with lung function. These results build on existing evidence of factors influencing lung function reported by Ostrowski and Barud in 2006 [18] among healthy adults. Sex, age, height, and weight are the most important predictors of lung function. Height and weight linearly correlate with the size of the lung, whereas age is a confounding factor. For ethnicity, by conducting a systematic review, variability, and differences between racial or ethnicity exist. Besides, the analysis from the present study confirms occupational factor was associated with all lung function parameters (p < 0.05). That is because the longer they are exposed to the traffic-related air pollutants, the possibility to have a decrement in lung function is high. As evidence to a significant association between average working hours and duration of services (years) with all lung function parameters, Gupta et al. [19] agreed that there is a significant decline in all spirometry parameters in traffic policemen in India as the duration of working is increased. While previous research has focused on sociodemographic and lifestyle factors, these results demonstrate that occupational factors also contribute to lung function. The data is essential and new findings that have not been done in a similar previous study among traffic police in Malaysia. It is novel in this present study that the results have not been reported in any studies regarding traffic police as the research among traffic police itself is limited. Other studies did not concern about other risk factors such as race, educational level, marital status, and smoking habits. Therefore, there is very limited literature on this topic.

Other than that, respondents who are working for more than five years have the odds of 0.16 having lung abnormalities of FVC value than those who are working for less than five years. It is similar to a study by Thippanna [20] conducted in India involving 665 traffic police personnel exposed to automobile pollutions in twin cities in India. These results are a new finding in Malaysia regarding occupational health among traffic policemen. Thus, from these findings, prediction models can be made as shown below;

The prediction model of having abnormal FVC value among traffic policemen is:

Logit (P) = In [P/1-P]

= 0.715 – [1.866*Duration of services]

The present study is important, in that numerous similar studies agree that the traffic police are having decrement in their lung function. Wongsurakiat et al. [21] studied lung function among 629 traffic policemen and 303 control groups also by using spirometry. The outcomes reported that the mean value of FEV1 and FVC of the traffic police were significantly lower than the control grouped. Thippanna [20] has investigated male traffic police constables by FVC, and 0.9% of them were found to have a severe restrictive ventilatory defect. Among them, 5.9% showed moderate, and another 18.6% shows a mild degree of respiratory restriction. Dutta T and Pal G [5] also found that a decrease in FEV1/FVC ratio among traffic policeman compared to controls. In Kuala Lumpur, Malaysia, Muhammad et al. 13], has found that traffic policeman was concluded as having a decrement in lung function parameters compared to the control group. Another comparative study among traffic police and general police in Selangor, Malaysia, by Muhammad N.S. et al. [1] also found that there are two times likely respondents among the exposed group has an abnormality in lung function status than control groups. Another study in Malaysia by Rasdi, [22] focusing on air pollution (PM_10_) with mental health (asthma-like respiratory symptoms) and among traffic police officers in urban and rural areas. The findings indicate that the level of PM_10_ significantly associated with the increased prevalence of a chronic cough and phlegm.

Nevertheless, the generalizability of the results is limited by a relatively small sample, and limited traffic police stations involved. Future research should direct attention to tackle and control the exposure of traffic police towards the factors influencing lung function. This line of research is crucial to developing our understanding of factors influencing lung function among traffic police and developing occupational safety and health policies aimed at protecting our traffic police personnel from occupational diseases. The data from this study can help by serving as a reference to the top management of traffic police officers to comply with the Occupational Safety and Health Act (OSHA, Act 514, 1994).

## Conclusions

From this study, occupational factors play a crucial role in the safety and health of traffic policemen, and these factors can be reconstructed to cater to their needs. The authorities should take action in generating flexible working hours and the duration of services accordingly. Conjointly, recognition of the hazards associated with occupational lung disease and prevention of exposure must be a high priority. As for recommendations, a physician such as occupational health doctors should be involved in the study so that it is easier to detect their respiratory problems. Besides, a long term follows up study is arguably the best research to do regarding the factors affecting pulmonary function and respiratory problems faced by the traffic police.
